# Study on the impact of air pollutants on childhood asthma in Nanjing based on a distributed lag non-linear model

**DOI:** 10.3389/fpubh.2025.1560896

**Published:** 2025-04-30

**Authors:** Wenjing Li, Ting Ding, Yahui Feng, Jin Xu, Zhuo Li

**Affiliations:** ^1^Department of Pharmacy, Children’s Hospital of Nanjing Medical University, Nanjing, China; ^2^National Climate Center, China Meteorological Administration, Beijing, China; ^3^Department of Emergency, Children’s Hospital of Nanjing Medical University, Nanjing, China

**Keywords:** air pollutants, childhood asthma, generalized additive model, distributed lag non-linear model, lag effect

## Abstract

**Background:**

Asthma, a chronic respiratory disease, is a significant public health concern globally. Air pollution has been identified as one of the key risk factors exacerbating respiratory conditions, particularly in children. Previous studies have shown a correlation between air pollution levels and asthma visits. However, the impact of air pollutants on childhood asthma visits stratified by gender, age and season remains a topic of interest, and further investigation is necessary to comprehend this complex relationship.

**Objective:**

This study aimed to explore the association between air pollution and childhood asthma visits in Nanjing from 2013 to 2021. It focused on the effects of various pollutants, including PM_2.5_, PM_10_, NO_2_, SO_2_, CO, and O_3_, and examined variations in impact based on demographic characteristics (gender and age) and seasonal changes.

**Methods:**

Data on childhood asthma visits and concentrations of air pollutants were collected and analyzed. The lag effects of pollutants on asthma visits were assessed, and the impacts were stratified by gender, age group (0–5 years, 6–11 years, and over 11 years), and season. Statistical methods were used to identify significant correlations and the timing of maximum effects of pollutants.

**Results:**

A positive correlation was found between childhood asthma visits and concentrations of AQI, PM_2.5_, PM_10_, SO_2_, NO_2_, and CO, with the strongest effects typically occurring on lag day 1. The study revealed that PM_2.5_ and PM_10_ had a more pronounced impact on females, and children aged 0–5 years were the most affected age group. Seasonal analysis showed that PM_2.5_ and SO_2_ had the greatest impact in spring, while PM_10_ and NO_2_ were most significant in winter. Notably, SO_2_ showed no significant impact on childhood over 11 years old or during summer, and the negative correlation between CO concentrations and childhood asthma visits in the summer, while in other seasons, the correlation was positive.

**Conclusion:**

The findings indicate a substantial effect of air pollutants, particularly PM_2.5_ and SO_2_, on childhood asthma, emphasizing the need for targeted pollution control measures. Variations in impact based on gender, age, and season suggest the importance of tailored interventions to protect vulnerable populations, especially young children in urban and industrial areas.

## 1 Introduction

Asthma is the most common chronic inflammatory airway disease in children and a major non-communicable disease affecting millions worldwide (1). Since the 1950s, the prevalence of childhood asthma has significantly increased, with current morbidity rates ranging from 3 to 7%, representing a 100% rise compared to a decade ago (2). In urban China, epidemiological studies have shown a similar trend, with asthma prevalence among children under 14 years old rising from 0.91% in 1990 to 3.02% in 2010, an increase of 50% per decade, reaching 4.9% in 2015 (3). This remains lower than the prevalence reported in developed countries, such as the United States (8.69%) (4), Australia (11.3%) (5), and the United Kingdom (15.4%) (6). However, the control rate of childhood asthma in China remains below one-third (7). In the eastern region of China, where Nanjing is located, the prevalence is relatively high due to rapid economic development and industrialization (8). This trend is particularly concerning, underscoring the urgent need for region-specific studies on asthma risk factors, especially air pollution.

The factors contributing to asthma and its progression are complex and remain under investigation. Key factors include allergies, genetic predisposition, infectious agents, and air pollution (9, 10). Although the exact causes of the rising asthma prevalence are not entirely clear, changes in environmental exposures are believed to play a significant role (11). Moreover, the impact of air pollutants varies depending on the weather and geographical conditions of different regions (12).

Over the past decade, rapid urbanization in China has led to deforestation, increased construction of large buildings, and a surge in the number of vehicles and plastics waste. Consequently, air pollutant concentrations have risen significantly, including suspended particulate matter with an aerodynamic diameter of less than 10 micrometers (PM_10_), fine particulate matter (PM_2.5_), sulfur dioxide (SO_2_), nitrogen dioxide (NO_2_), carbon monoxide (CO), and ozone (O_3_). These pollutants are all potential risk factors for childhood asthma (13–15). Since 2013, real-time monitoring of PM_2.5_ in various cities has introduced new indices for air quality evaluation, including PM_2.5_, CO, and O_3_. In urban and industrialized areas, an Air Quality Index (AQI) between 201 and 250 is considered unhealthy (16). Given these developments, investigating the effects of air pollution on children’s health, particularly childhood asthma in Chinese cities, has become increasingly relevant and necessary.

Several studies in major Chinese cities have explored the relationship between PM_2.5_ exposure and adverse health outcomes, with results aligning with findings from developed countries (17–20). A study on asthma-related hospital visits among children aged 0–18 years in Chongqing indicated that short-term exposure to PM10, PM2.5, carbon dioxide, nitrogen dioxide, and carbon monoxide could trigger asthma hospitalizations, with nitrogen dioxide playing a particularly significant role (15). Additionally, research in Shanghai found a significant association between childhood asthma hospitalizations and ambient concentrations of black carbon (BC) and PM2.5 (21). However, few studies have comprehensively examined the exposure-lag response relationship of multiple air pollutants on childhood asthma, and no such research has been conducted in Nanjing. Time series models, including case-crossover designs and distributed lag nonlinear models (DLNM), are commonly used to assess asthma risks associated with air pollution in different urban environments. These methods have been widely applied in developed countries to establish short-term associations between air pollutants and acute asthma exacerbations. Expanding such studies in China, particularly in highly urbanized areas, is crucial for understanding the susceptibility of specific populations and informing targeted public health interventions.

To address this gap, this study aims to investigate the exposure-lag-response relationship between major air pollutant concentrations and childhood asthma visits in Nanjing, China. As an epidemiological investigation, we conducted stratified analyses based on age, gender and season to assess the impact of various parameters on asthma visits. The findings provide a scientific basis for early research on the etiology of childhood asthma and the early risk warning of air pollution.

## 2 Materials and methods

### 2.1 Study design and participants

#### 2.1.1 Study location

This study was conducted in Nanjing, the capital city of Jiangsu Province in Southeast China. Nanjing is situated on the vast plain of the lower reaches of the Yangtze River, with geographical coordinates of approximately at 32°03’ N latitude and 118°47′E longitude. Covering an area of 6587.02 square kilometers, Nanjing had a population of 9.42 million in 2022. The city experiences a northern subtropical monsoon climate, marked by four distinct seasons. The annual average temperature is around 16°C, and the yearly precipitation is approximately 1,160 mm. The frost-free period in Nanjing lasts about 237 days.

#### 2.1.2 Study group

The Children’s Hospital of Nanjing Medical University, the only specialized pediatric hospital in Nanjing, serves both local residents and individuals from surrounding regions. Clinical data for this study were extracted from the hospital’s computerized database, which includes demographic details such as age, gender, and date of hospital visits.

Specifically, we collected data on daily outpatient and emergency visits of individuals under the age of 18 who had been diagnosed with asthma, status asthmatics, bronchitis asthma, or cough variant asthma by qualified pediatricians. The diagnosis of asthma was based on the Global Initiative Asthma (GINA) 2024 guidelines (22). Cases where asthma was not the primary diagnosis but was accompanied by other infectious diseases, such as pneumonia, were excluded. The data collection period spanned from January 1, 2013, to December 31, 2021.

#### 2.1.3 Data on air pollution and the AQI

Daily air pollution data, including PM_10_, PM_2.5_, SO_2_, NO_2_, CO, O_3_, and the AQI for Nanjing, temperature and humidity data, were obtained from the National Climate Center, China Meteorological Administration, for the period from January 1, 2013, to December 31, 2021.

O3 levels were assessed based on the maximum 8-h average concentration within a day, while the concentrations of the other five pollutants (PM_10_, PM_2.5_, SO_2_, NO_2_, CO) were evaluated using their 24-h average values. The AQI is a composite index derived from the levels of these six atmospheric pollutants. The AQI standard in China is similar to that in the United States and is categorized as follows: excellent (0–50), good (51–100), lightly polluted (101–150), moderately polluted (151–200), heavily polluted (201–300), and hazardous (>300) (23). In this study, the AQI was used as an indicator of the cumulative effect of air pollution on childhood asthma.

#### 2.1.4 Study design and statistical analysis

In our study, the DLNM was used to evaluate the exposure-response relationship between air pollutant concentrations and childhood asthma visits, exploring whether air pollution has immediate and lagged effects on childhood asthma visits. The GAM was chosen as the baseline model. Given that childhood asthma visits are relatively rare in the general population, a Poisson distribution was used as the link function in the model. To account for the potential influence of meteorological factors on visit rates, spline smoothing functions were applied to control for temperature and relative humidity in the regression model between air pollution and asthma visits. Additionally, confounding parameters were introduced to adjust for the effects of weekdays and holidays. The correlation between pollutants and meteorological factors was assessed using Spearman correlation analysis.

The study period was divided into four-week (28-day) time strata. Within each stratum, the day of the week corresponding to the asthma visit was designated as the case day, while the same days of the week in the other 3 weeks within the same stratum were used as control days. This design is equivalent to a stratified case–control study, allowing for a comparison of air pollutant exposure on case days with that on control days. To explore the association between air pollutant exposure and the risk of asthma-related hospital visits, we used a combination of the distributed lag non-linear model (DLNM) design. This approach enabled us to examine both the lagged and non-linear effects of air pollutants on asthma visit risk.

We examined the associations between age, gender, season of illness onset, and exposure to air pollutants. After removing duplicate and missing data, we extracted the daily counts of asthma attacks in children aged 0 to 18 years from January 1, 2013, to December 31, 2021. The age groups were categorized as 0–5 years, 6–11 years, and over 11 years. Each year was divided into four seasons: spring (March to May), summer (June to August), autumn (September to November), and winter (December to February). Weekends and public holidays, including New Year’s Day, the Spring Festival, Tomb-sweeping Day, International Worker’s Day, the Dragon Boat Festival, the Mid-Autumn Festival, and National Days, were identified as holidays.

We constructed basic models for each specified pollutant, adjusting for the effects of the remaining pollutants using spline function. The resulting GAM equation was as follows:

$\begin{array}{l} \log\left[E\left(Y_{t}\right)\right]=\alpha +\beta \times C_{\mathrm{pollutant}}+\mathrm{ns}\left(\mathrm{time,df}\right)+\\ \mathrm{ns}\left(\mathrm{temperature,df}\right)+\mathrm{ns}\left(\mathrm{humidity,df}\right)+\\ as.\mathrm{factor}\left(D\right) \end{array}$ .

In this equation, Log[E(Y_t_)] represents the natural logarithm of the expected value of the total daily visits for childhood asthma on day t. α is the residual value, and β is the regression coefficient for the air pollutant concentration (C_pollutant_) on day t. The model includes non-parametric spline smoothing functions, denoted by ns (), which control for the effects of time, temperature, and humidity. The degrees of freedom (df) for these smoothing functions were determined using the model with the smallest Akaike Information Criterion (AIC). In our study, the degrees of freedom for time, temperature, and humidity were set to 7, 3, and 3, respectively. The variable D is a categorical variable that accounts for weekdays, holidays, and seasonal dummy variables. It is included in the model as as.factor (D) to adjust for the effects of weekdays, holidays, and different seasons. To estimate the lag effect and cumulative relative risk (RR), we examined the impact of a 10 ug/m3 increase in the concentration of each pollutant and the AQI on the number of asthma visits across various lag intervals, ranging from 0 to 10 days. We then calculated the distribution of lag effects and cumulative RRs and plotted them accordingly. Furthermore, we performed stratified analyses by age, gender, and season to calculate the RRs for different groups.

Categorical variables were presented as frequencies and proportions, while continuous variables were expressed as medians and interquartile ranges (IQR). All statistical analyses were conducted using R statistical software version 4.1.3. A two-sided *p*-value of less than 0.05 was considered statistically significant.

## 3 Results

### 3.1 Descriptive analysis

From January 1, 2013, to December 31, 2021, a total of 248,996 childhood asthma visits were recorded over 3,077 days. Among these visits, 163,167 (65.53%) were boys and 85,829 (34.47%) were girls, resulting in a male-to-female ratio of 1.901: 1. The age distribution of childhood asthma visits revealed that 55.50% (138,183) were in the 0–5 years age group, 39.25% (97,725) were in the 6–11 years age group, and 5.26% (13,088) were over 11 years old, showing a significant difference (*p* < 0.001).

In terms of seasonal distribution, the highest number of hospital visits for childhood asthma occurred in autumn, followed by summer and winter, while spring had the lowest number of visits. Throughout the study period, the average daily temperature was (16.67 ± 8.99) °C, and the average relative humidity was (72.50 ± 14.61) %. The daily mean concentrations of pollutants from 2013 to 2021 were as follows: PM_2.5_ (50.87 ± 36.69 ug/m^3^), PM_10_ (89.99 ± 55.48 ug/m^3^), O_3_ (104.55 ± 46.99 ug/m^3^), SO_2_ (16.58 ± 13.76 ug/m^3^), NO_2_ (45.02 ± 19.63 ug/m^3^), and CO (0.90 ± 0.35 ug/m^3^). The average concentrations of PM_2.5_, PM_10_, and NO_2_ exceeded the national air quality standard of Grade II, whereas the average concentrations of SO_2_, O_3_ and CO were below the Grade II threshold. The average AQI during the study period was (79.61 ± 43.22), indicating overall good air quality. Table 1 provides a comprehensive summary of the statistical characteristics of the 248,996 childhood asthma visits, along with meteorological factors, air quality indices, and pollutant concentrations in Nanjing, China.

**Table 1:** Summary statistics of childhood asthma visits, meteorological factors, air quality and pollutants in Nanjing.

Characteristics	Mean ± SD	Minimum	First quartile	Median	Third quartile	Maximum	IQR	N (%)	*p*
Daily asthma visits count								248,996(100.0)	
Age group									<0.001
0–5 years	22.45 ± 16.89	0.00	10.00	19.00	30.00	191.00	20.00	138,183(55.50)	
6–11 years	15.88 ± 11.45	0.00	8.00	13.00	22.00	105.00	14.00	97,725(39.25)	
Over 11 years	2.13 ± 2.49	0.00	0.00	1.00	3.00	22.00	3.00	13,088(5.26)	
Gender									<0.001
Boys	17.68 ± 17.00	0.00	3.00	14.00	28.00	191.00	25.00	163,167(65.53)	
Girls	9.30 ± 10.05	0.00	1.00	7.00	14.00	158.00	13.00	85,829(34.47)	
Illness onset season									<0.001
Spring	11.79 ± 12.82	0.00	2.00	8.00	18.00	156.00	16.00	56,952(22.87)	
Summer	13.83 ± 13.43	0.00	3.00	10.00	21.00	105.00	18.00	61,146(24.56)	
Autumn	15.21 ± 15.80	0.00	2.00	10.00	23.00	93.00	21.00	68,793(27.63)	
Winter	13.25 ± 15.84	0.00	2.00	8.00	20.00	191.00	18.00	62,105(24.94)	
Meteorological factors									
Temperature (°C)	16.67 ± 8.99	−6.70	8.80	17.20	24.30	34.73	15.50	–	–
Relative humidity (%)	72.50 ± 14.61	28.30	62.80	73.00	84.00	100.00	21.20	–	–
Air quality and pollutants									
AQI	79.61 ± 43.22	14.42	51.22	69.88	95.31	378.27	44.09	–	–
PM_2.5_ (ug/m^3^)	50.87 ± 36.69	5.02	25.67	40.33	65.28	332.08	39.61	–	–
PM_10_ (ug/m^3^)	89.99 ± 55.48	7.72	50.72	77.00	115.64	443.71	64.92	–	–
O_3_ (ug/m^3^)	104.55 ± 46.99	8.11	67.44	99.06	134.89	280.00	67.44	–	–
SO_2_ (ug/m^3^)	16.58 ± 13.76	2.85	7.65	12.47	20.82	139.64	13.18	–	–
NO_2_ (ug/m^3^)	45.02 ± 19.63	5.58	30.73	41.19	55.93	141.62	25.20	–	–
CO (ug/m^3^)	0.903 ± 0.348	0.268	0.670	0.825	1.051	3.106	0.381	–	–

*p < 0.05.

**Figure 1:**
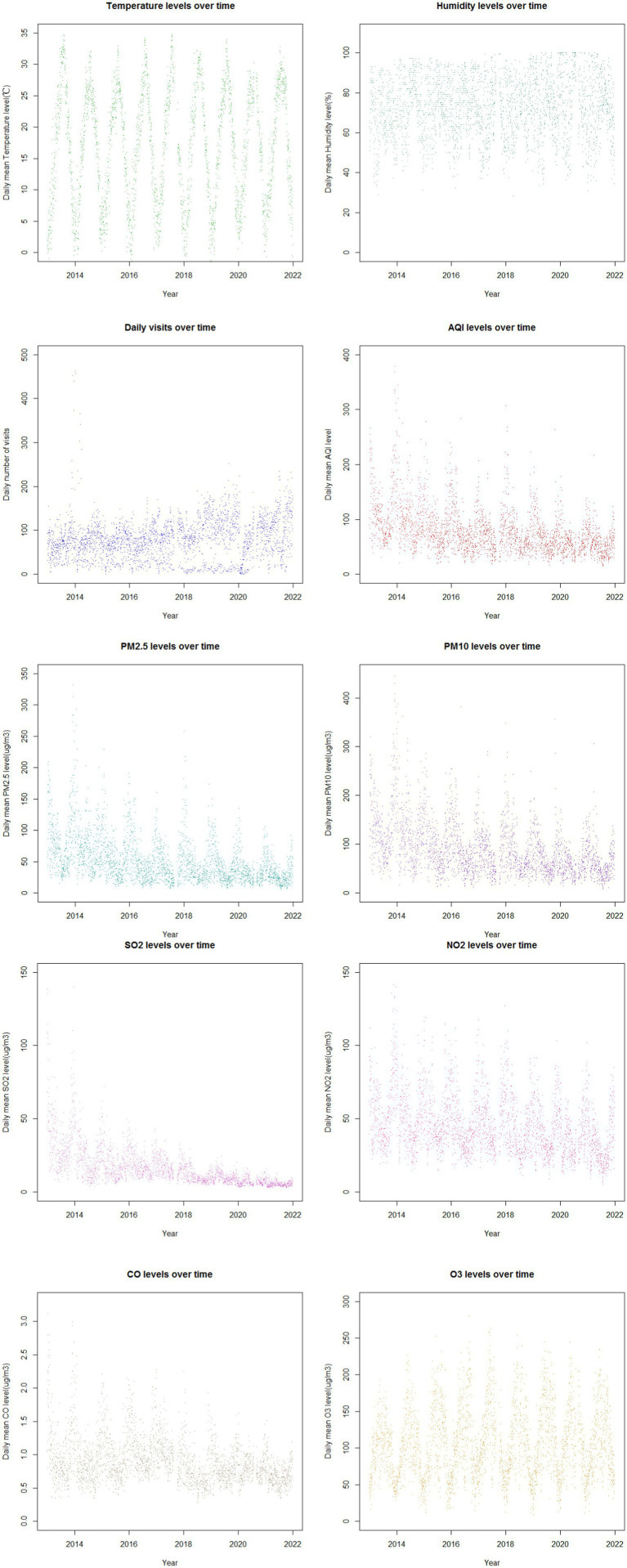
The time trends of the daily childhood asthma visits with asthma, concentrations of major pollutants and meteorological factors in Nanjing from 2013 to 2021.

Figure 1 illustrates the time trends of daily childhood asthma visits, pollutant concentrations, and meteorological factors in Nanjing throughout the study period. Temperature and relative humidity exhibited periodic fluctuations with overall stable trends. The number of childhood asthma visits increased annually from 2013 to 2021, with a more pronounced rise starting in 2018. Notably, visits peaked in January and October–December, reaching the highest points in December 2013 and January 2014. The concentrations of PM_2.5_, PM_10_, SO_2_, NO_2_ and CO displayed similar periodic variation trends as asthma visits. These pollutants showed a general decline over the years, with the highest levels recorded in December 2013 and January 2014. The most significant reduction was observed in SO_2_ concentrations, while other pollutants experienced a more substantial decline beginning in 2020. Additionally, the concentrations of air pollutants, except for O_3_ exhibited clear seasonality, with highest levels in the cold season compared to the warm season. The highest concentrations were observed in January and December, while the lowest concentrations occurred in June, July, and August. In contrast, O_3_ concentrations were higher in the warm season compared to the cold season. Temperature demonstrated obvious seasonal trends, whereas relative humidity did not show significant seasonality.

### 3.2 Correlation between air pollutants and meteorological factors

**Figure 2:**
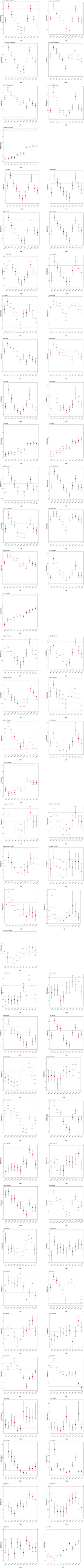
Lag effect of air pollutants concentration on daily visits with childhood asthma for total population and different age, different gender and different season group in Nanjing from 2013 to 2021.

Table 2 presents the Spearman correlation coefficients, showing the relationships between air pollutant concentrations and meteorological factors in Nanjing from 2013 to 2021. A visual representation of these correlation coefficients is provided in Figure 2. The analysis revealed that, with the exception of O_3_, there was a significant positive correlation between the concentrations of various air pollutants, including PM_2.5_, PM_10_, SO_2_, NO_2_, and CO. In contrast, temperature and relative humidity were significantly negatively correlated with the concentrations of these air pollutants. Additionally, a significant positive correlation was found between temperature and O_3_, while a significant negative correlation was observed between temperature and other pollutants such as SO_2,_ NO_2_, CO and O_3_, as well as relative humidity. Moreover, a strong correlation was observed between PM_10_ and PM_2.5_ (*r* = 0.917, *p* < 0.01), indicating a close association between these two particulate matter pollutants. On the other hand, there was little correlation between SO_2_ and O_3_ (*r* = 0.008, *p* > 0.05), suggesting a weak relationship between these two pollutants.

**Table 2:** Spearman correlation coefficient between air pollutant concentration and meteorological factors in Nanjing from 2013 to 2021.

Characteristics	AQI	PM_2.5_	PM_10_	O_3_	SO_2_	NO_2_	CO	Temperature	Relative humidity
AQI	1.000	0.948*	0.976*	−0.040*	0.685*	0.706*	0.662*	−0.294*	−0.323*
PM_2.5_		1.000	0.917*	−0.150*	0.628*	0.687*	0.681*	−0.354*	−0.146*
PM_10_			1.000	−0.067*	0.713*	0.751*	0.644*	−0.319*	−0.384*
O_3_				1.000	0.008	−0.206*	−0.139*	0.658*	−0.277*
SO_2_					1.000	0.627*	0.573*	−0.129*	−0.407*
NO_2_						1.000	0.616*	−0.389*	−0.306*
CO							1.000	−0.247*	−0.063*
Temperature								1.000	0.120*
Relative humidity									1.000

*p < 0.05.

### 3.3 Lag effect of air pollutants on daily visits for childhood asthma

#### 3.3.1 Lag effect in the total population

Table 3 show the association between the AQI, air pollutant concentrations, and the number of childhood asthma visits in Nanjing from 2013 to 2021, considering different lag days. Among the air pollutants analyzed (PM_2.5_, PM_10_, SO_2_, NO_2_, and CO), all except O_3_ displayed similar effects on daily childhood asthma visits across different lag days. The effects peaked on lag day 1 and showed a secondary peak on lag day 8, whereas O_3_ had its maximum impact on lag day 10.

**Table 3:** Relative risks (RRs) and 95% confidence intervals (CI) for association between air pollutant concentration and childhood asthma visits in total population in different lag days in Nanjing from 2013 to 2021.

Lag days/d	AQI	PM_2.5_	PM_10_	O_3_	SO_2_	NO_2_	CO
0	1.0058(1.0047–1.0069)*	1.0060(1.0046–1.0073)*	1.0059(1.0050–1.0068)*	0.9866(0.9853–0.9879)*	1.0426(1.0380–1.0472)*	1.0344(1.0319–1.0370)*	1.9849(1.7371–2.2681)*
1	1.0109(1.0098–1.0120)*	1.0126(1.0112–1.0139)*	1.0095(1.0086–1.0104)*	0.9872(0.9860–0.9883)*	1.0546(1.0503–1.0589)*	1.0437(1.0412–1.0462)*	4.3131(3.7844–4.9156)*
2	1.0079(1.0068–1.0090)*	1.0084(1.0071–1.0098)*	1.0073(1.0064–1.0081)*	0.9879(0.9868–0.9890)*	1.0490(1.0447–1.0532)*	1.0392(1.0367–1.0416)*	3.2038(2.8082–3.6551)*
3	1.0058(1.0047–1.0069)*	1.0063(1.0049–1.0076)*	1.0059(1.0051–1.0068)*	0.9879(0.9868–0.9890)*	1.0409(1.0367–1.0451)*	1.0359(1.0334–1.0383)*	1.9969(1.7471–2.2825)*
4	1.0034(1.0023–1.0045)*	1.0033(1.0020–1.0047)*	1.0036(1.0027–1.0045)*	0.9906(0.9895–0.9917)*	1.0353(1.0310–1.0396)*	1.0318(1.0294–1.0342)*	1.7325(1.5134–1.9834)*
5	1.0020(1.0008–1.0031)*	1.0006(0.9992–1.0020)*	1.0022(1.0013–1.0031)*	0.9908(0.9898–0.9919)	1.0211(1.0167–1.0255)*	1.0263(1.0239–1.0287)*	1.3514(1.1784–1.5498)*
6	1.0025(1.0013–1.0036)*	0.9996(0.9982–1.0010)*	1.0029(1.0020–1.0038)*	0.9910(0.9899–0.9921)	1.0386(1.0342–1.0431)*	1.0289(1.0265–1.0313)*	1.7176(1.4961–1.9720)*
7	1.0064(1.0053–1.0076)*	1.0057(1.0043–1.0071)*	1.0055(1.0046–1.0064)*	0.9961(0.9950–0.9971)*	1.0411(1.0366–1.0455)*	1.0231(1.0207–1.0256)*	2.4241(2.1100–2.7851)*
8	1.0101(1.0090–1.0112)*	1.0109(1.0095–1.0123)*	1.0087(1.0079–1.0096)*	0.9958(0.9947–0.9969)*	1.0455(1.0412–1.0498)*	1.0315(1.0290–1.0339)*	3.7994(3.3126–4.3577)*
9	1.0060(1.0048–1.0071)*	1.0057(1.0043–1.0071)*	1.0064(1.0055–1.0073)*	0.9967(0.9956–0.9978)*	1.0393(1.0350–1.0436)*	1.0272(1.0248–1.0297)*	2.4180(2.1060–2.7761)*
10	1.0050(1.0039–1.0062)*	1.0045(1.0031–1.0059)*	1.0054(1.0045–1.0063)*	0.9969(0.9958–0.9980)*	1.0323(1.0279–1.0367)*	1.0248(1.0223–1.0273)*	2.0793(1.8094–2.3895)*

*p < 0.05.

Among these pollutants, the increase in CO concentration had the most significant impact on the number of visits. When the concentrations of PM_2.5_, PM_10_, SO_2_, and NO_2_ increased by 10 ug/m^3^, the number of daily asthma visits in children increased by 1.26% (95% CI: 1.0112–1.0139), 0.95% (95% CI: 1.0086–1.0104), 5.46% (95% CI: 1.0503–1.0589), and 4.37% (95% CI: 1.0412–1.0462), respectively, on lag day 1, with statistical significance. At the secondary peak on lag day 8, a 10 ug/m^3^ increase in PM_2.5_, PM_10_, SO_2_ and NO_2_, was associated with a 1.09% (95% CI: 1.0095–1.0123), 0.87% (95% CI: 1.0079–1.0096), 4.55% (95% CI: 1.0412–1.0498), and 3.15% (95% CI: 1.0290–1.0339) increase in daily asthma visits, respectively, all statistically significant.

#### 3.3.2 Lag effect by gender

Tables 4, 5, show the association between the AQI, air pollutant concentrations, and the number of childhood asthma visits, stratified by gender and lag days in Nanjing from 2013 to 2021. The effects of AQI and air pollutants (PM_2.5_, PM_10_, SO_2_ NO_2_, and CO) excluding O_3_, on daily childhood asthma visits exhibited similar patterns across genders, consistent with the total population analysis. The maximum impact was observed on lag day 1, with a secondary peak on lag day 8, and the increase in CO concentration had the most significant impact on the number of visits. Notably, for female asthma visits, PM_10_ demonstrated the most substantial lag effect on both lag days 1 and 8.

**Table 4:** Relative risks (RRs) and 95% confidence intervals (CI) for association between air pollutant concentration and childhood asthma visits for male children in different lag days in Nanjing from 2013 to 2021.

Lag days/d	AQI	PM_2.5_	PM_10_	O_3_	SO_2_	NO_2_	CO
0	1.0057(1.0043–1.0071)*	1.0057(1.0041–1.0074)*	1.0058(1.0047–1.0069)*	0.9871(0.9855–0.9887)*	1.0355(1.0299–1.0412)*	1.0333(1.0301–1.0364)*	1.8647(1.5810–2.1994)*
1	1.0096(1.0083–1.0110)*	1.0108(1.0092–1.0125)*	1.0084(1.0074–1.0095)*	0.9881(0.9866–0.9895)*	1.0465(1.0412–1.0519)*	1.0418(1.0388–1.0449)*	3.6811(3.1299–4.3294)*
2	1.0066(1.0052–1.0079)*	1.0067(1.0051–1.0084)*	1.0062(1.0052–1.0073)*	0.9885(0.9871–0.9899)*	1.0388(1.0336–1.0440)*	1.0375(1.0345–1.0405)*	2.7255(2.3144–3.2098)*
3	1.0046(1.0032–1.0060)*	1.0049(1.0032–1.0066)*	1.0049(1.0039–1.0060)*	0.9875(0.9861–0.9888)*	1.0317(1.0265–1.0369)*	1.0362(1.0332–1.0392)*	1.8206(1.5427–2.1486)*
4	1.0019(1.0005–1.0033)*	1.0017(1.0000–1.0034)*	1.0023(1.0012–1.0034)	0.9908(0.9894–0.9921)*	1.0262(1.0209–1.0315)*	1.0300(1.0270–1.0329)*	1.4130(1.1946–1.6713)*
5	1.0006 (0.9992–1.0020)	0.9991(0.9974–1.0008)	1.0010(0.9999–1.0021)	0.9901(0.9888–0.9914)*	1.0135(1.0081–1.0189)*	1.0236(1.0207–1.0266)*	1.1431(0.9645–1.3548)
6	1.0018(1.0004–1.0033)*	0.9992(0.9975–1.0010)	1.0021(1.0010–1.0032)*	0.9911(0.9897–0.9924)*	1.0293(1.0238–1.0348)*	1.0264(1.0235–1.0294)*	1.5383(1.2964–1.8252)*
7	1.0049(1.0035–1.0064)*	1.0041(1.0024–1.0059)*	1.0039(1.0028–1.0050)*	0.9960(0.9947–0.9973)*	1.0309(1.0254–1.0364)*	1.0211(1.0181–1.0241)*	2.1165(1.7821–2.5136)*
8	1.0086(1.0072–1.0100)*	1.0093(1.0076–1.0110)*	1.0073(1.0062–1.0084)*	0.9959(0.9945–0.9972)*	1.0374(1.0321–1.0428)*	1.0280(1.0250–1.0310)*	3.1387(2.6478–3.7206)*
9	1.0046(1.0032–1.0060)*	1.0040(1.0023–1.0057)*	1.0051(1.0039–1.0062)*	0.9965(0.9952–0.9979)*	1.0288(1.0235–1.0341)*	1.0240(1.0210–1.0271)*	1.9066(1.6063–2.2630)*
10	1.0037(1.0023–1.0051)*	1.0032(1.0014–1.0049)*	1.0042(1.0031–1.0053)*	0.9960(0.9947–0.9973)*	1.0223(1.0169–1.0278)*	1.0217(1.0187–1.0248)*	1.7081(1.4377–2.0294)*

*p < 0.05.

**Table 5:** Relative risks (RRs) and 95% confidence intervals (CI) for association between air pollutant concentration and childhood asthma visits for female children in different lag days in Nanjing from 2013 to 2021.

Lag days/d	AQI	PM_2.5_	PM_10_	O_3_	SO_2_	NO_2_	CO
0	1.0060(1.0040–1.0079)*	1.0065(1.0041–1.0088)*	1.0061(1.0046–1.0077)*	0.9855(0.9833–0.9877)*	1.0556(1.0479–1.0633)*	1.0363(1.0320–1.0405)*	2.2263(1.7756–2.7914)*
1	1.0134(1.0115–1.0152)*	1.0158(1.0136–1.0181)*	1.0115(1.0100–1.0129)*	0.9854(0.9834–0.9874)*	1.0694(1.0622–1.0767)*	1.0471(1.0429–1.0513)*	5.7956(4.6467–7.2285)*
2	1.0104(1.0086–1.0123)*	1.0117(1.0094–1.0139)*	1.0094(1.0079–1.0108)*	0.9868(0.9849–0.9887)*	1.0675(1.0604–1.0746)*	1.0421(1.0380–1.0462)*	4.3232(3.4607–5.4006)*
3	1.0082(1.0063–1.0100)*	1.0089(1.0066–1.0112)*	1.0078(1.0064–1.0093)*	0.9887(0.9869–0.9906)*	1.0579(1.0508–1.0650)*	1.0354(1.0314–1.0395)*	2.3667(1.8875–2.9674)*
4	1.0063(1.0044–1.0082)*	1.0064(1.0041–1.0087)*	1.0060(1.0045–1.0075)*	0.9904(0.9885–0.9922)*	1.0520(1.0448–1.0593)*	1.0353(1.0313–1.0394)*	2.5238(2.0090–3.1705)*
5	1.0045(1.0026–1.0064)*	1.0033(1.0010–1.0057)*	1.0046(1.0031–1.0062)*	0.9923(0.9905–0.9942)*	1.0353(1.0279–1.0427)*	1.0313(1.0272–1.0354)*	1.8397(1.4598–2.3186)*
6	1.0037(1.0017–1.0056)*	1.0004(0.9980–1.0027)	1.0045(1.0030–1.0060)*	0.9908(0.9890–0.9927)*	1.0561(1.0485–1.0637)*	1.0334(1.0293–1.0375)*	2.1140(1.6733–2.6709)*
7	1.0092(1.0073–1.0111)*	1.0085(1.0062–1.0109)*	1.0084(1.0068–1.0099)*	0.9962(0.9944–0.9981)*	1.0600(1.0524–1.0675)*	1.0266(1.0225–1.0307)	3.1189(2.4658–3.9449)*
8	1.0129(1.0110–1.0148)*	1.0140(1.0117–1.0164)*	1.0115(1.0100–1.0130)*	0.9958(0.9939–0.9977)*	1.0602(1.0529–1.0675)*	1.0379(1.0337–1.0421)*	5.3918(4.2775–6.7963)*
9	1.0085(1.0066–1.0105)*	1.0088(1.0064–1.0111)*	1.0090(1.0075–1.0105)*	0.9971(0.9952–0.9989)*	1.0587(1.0514–1.0659)*	1.0334(1.0292–1.0375)*	3.7326(2.9570–4.7116)*
10	1.0075(1.0056–1.0094)*	1.0069(1.0045–1.0093)*	1.0077(1.0062–1.0092)*	0.9987(0.9968–1.0005)	1.0508(1.0434–1.0583)*	1.0306(1.0265–1.0348)*	2.9778(2.3543–3.7662)*

*p < 0.05.

For male children, when the concentrations of PM_2.5_, PM_10_, SO_2_, and NO_2_ increased by 10 ug/m^3^, the number of daily asthma visits increased by 1.08% (95% CI: 1.0092–1.0125), 0.84% (95% CI: 1.0074–1.0095), 4.65% (95% CI: 1.0412–1.0519), and 4.18% (95% CI: 1.0388–1.0449), respectively, on lag day 1, with statistical significance. For female children, a 10 ug/m^3^ increase in the concentrations of PM_2.5_, SO_2_, and NO_2_ on lag day 1 was associated with increases in daily asthma visits by 1.58% (95% CI: 1.0136–1.0181), 6.94% (95% CI: 1.0622–1.0767), and 4.71% (95% CI: 1.0429–1.0513), respectively, all statistically significant. Additionally, a 10 ug/m^3^ increase in PM_10_ resulted in a 1.15% (95% CI: 1.0100–1.0129) increase in daily asthma visits for females on both lag days 1 and 8, with statistical significance.

#### 3.3.3 Lag effect by age

Tables 6–8 present the association between AQI, air pollutant concentrations, and the number of childhood asthma visits stratified by age and lag days in Nanjing from 2013 to 2021.

**Table 6:** Relative risks (RRs) and 95% confidence intervals (CI) for association between air pollutant concentration and childhood asthma visits for 0–5 years old children in different lag days in Nanjing from 2013 to 2021.

Lag days/d	AQI	PM_2.5_	PM_10_	O_3_	SO_2_	NO_2_	CO
0	1.0079(1.0064–1.0094)*	1.0075(1.0057–1.0094)*	1.0084(1.0071–1.0096)*	0.9836(0.9819–0.9854)*	1.0571(1.0511–1.0631)*	1.0406(1.0372–1.0440)*	2.2337(1.8689–2.6697)*
1	1.0146(1.0132–1.0161)*	1.0163(1.0146–1.0181)*	1.0127(1.0116–1.0139)*	0.9876(0.9860–0.9892)*	1.0681(1.0625–1.0737)*	1.0512(1.0479–1.0545)*	6.0091(5.0508–7.1494)*
2	1.0121(1.0107–1.0136)*	1.0131(1.0113–1.0148)*	1.0107(1.0096–1.0119)*	0.9883(0.9867–0.9898)*	1.0684(1.0629–1.0740)*	1.0488(1.0456–1.0521)*	5.1188(4.3002–6.0931)*
3	1.0098(1.0083–1.0112)*	1.0107(1.0089–1.0125)*	1.0094(1.0083–1.0105)*	0.9886(0.9871–0.9900)*	1.0567(1.0512–1.0623)*	1.0467(1.0434–1.0500)*	3.0703(2.5726–3.6643)*
4	1.0069(1.0054–1.0084)*	1.0068(1.0050–1.0086)*	1.0070(1.0058–1.0082)*	0.9911(0.9897–0.9926)*	1.0518(1.0461–1.0575)*	1.0430(1.0398–1.0463)*	2.7269(2.2799–3.2615)*
5	1.0058(1.0043–1.0073)*	1.0046(1.0028–1.0065)*	1.0055(1.0043–1.0067)*	0.9919(0.9904–0.9933)*	1.0331(1.0272–1.0390)*	1.0377(1.0344–1.0409)*	1.9308(1.6098–2.3158)*
6	1.0053(1.0038–1.0068)*	1.0021(1.0002–1.0039)	1.0055(1.0043–1.0067)*	0.9910(0.9895–0.9925)*	1.0552(1.0492–1.0613)*	1.0393(1.0361–1.0426)*	2.2304(1.8551–2.6817)*
7	1.0091(1.0076–1.0107)*	1.0079(1.0060–1.0097)*	1.0087(1.0075–1.0099)*	0.9947(0.9932–0.9962)*	1.0613(1.0553–1.0673)*	1.0349(1.0316–1.0381)	3.1195(2.5900–3.7574)*
8	1.0116(1.0101–1.0131)*	1.0121(1.0102–1.0139)*	1.0108(1.0096–1.0120)*	0.9960(0.9945–0.9975)*	1.0628(1.0571–1.0685)*	1.0436(1.0403–1.0469)*	4.5865(3.8186–5.5088)*
9	1.0077(1.0061–1.0092)*	1.0070(1.0051–1.0089)*	1.0082(1.0070–1.0094)*	0.9975(0.9960–0.9990)*	1.0571(1.0514–1.0628)*	1.0387(1.0354–1.0420)*	2.6937(2.2395–3.2399)*
10	1.0073(1.0057–1.0088)*	1.0066(1.0047–1.0084)*	1.0076(1.0065–1.0088)*	0.9984(0.9969–0.9999)*	1.0554(1.0496–1.0613)*	1.0378(1.0345–1.0411)*	2.6895(2.2340–3.2378)*

*p < 0.05.

**Table 7:** Relative risks (RRs) and 95% confidence intervals (CI) for association between air pollutant concentration and childhood asthma visits for 6–11 years old children in different lag days in Nanjing from 2013 to 2021.

Lag days/d	AQI	PM_2.5_	PM_10_	O_3_	SO_2_	NO_2_	CO
0	1.0039(1.0021–1.0057)*	1.0048(1.0026–1.0070)*	1.0031(1.0017–1.0046)*	0.9905(0.9885–0.9926)*	1.0275(1.0200–1.0349)*	1.0279(1.0238–1.0320)*	1.8410(1.4867–2.2797)*
1	1.0061(1.0043–1.0079)*	1.0076(1.0055–1.0098)*	1.0050(1.0036–1.0065)*	0.9873(0.9855–0.9891)*	1.0384(1.0314–1.0455)*	1.0342(1.0303–1.0382)*	2.8768(2.3276–3.5555)*
2	1.0025(1.0007–1.0043)*	1.0025(1.0004–1.0047)*	1.0027(1.0012–1.0041)*	0.9881(0.9864–0.9899)*	1.0253(1.0185–1.0323)*	1.0270(1.0231–1.0309)*	1.8303(1.4767–2.2687)*
3	1.0014(0.9996–1.0032)	1.0015(0.9993–1.0037)	1.0018(1.0004–1.0032)*	0.9878(0.9861–0.9895)*	1.0227(1.0159–1.0296)*	1.0234(1.0195–1.0273)*	1.3032(1.0485–1.6197)*
4	1.0000(0.9982–1.0018)	1.0000(0.9978–1.0022)*	1.0000(0.9985–1.0014)	0.9908(0.9891–0.9924)*	1.0164(1.0095–1.0234)*	1.0197(1.0157–1.0236)*	1.1303(0.9072–1.4081)
5	0.9978(0.9960–0.9997)*	0.9959(0.9937–0.9982)*	0.9986(0.9972–1.0001)	0.9903(0.9886–0.9920)*	1.0084(1.0014–1.0154)*	1.0128(1.0088–1.0167)*	0.9634(0.7716–1.2029)
6	0.9996(0.9978–1.0015)	0.9972(0.9949–0.9994)*	1.0002(0.9987–1.0016)	0.9915(0.9898–0.9932)*	1.0199(1.0128–1.0271)*	1.0167(1.0128–1.0207)*	1.3396(1.0723–1.6736)*
7	1.0035(1.0017–1.0053)*	1.0035(1.0012–1.0057)*	1.0018(1.0004–1.0033)*	0.9986(0.9969–1.0003)	1.0203(1.0132–1.0275)*	1.0101(1.0062–1.0141)*	1.9804(1.5850–2.4744)*
8	1.0086(1.0068–1.0104)*	1.0098(1.0075–1.0120)*	1.0065(1.0051–1.0080)*	0.9965(0.9948–0.9982)*	1.0255(1.0185–1.0325)*	1.0175(1.0136–1.0215)*	3.2184(2.5811–4.0129)*
9	1.0045(1.0027–1.0064)*	1.0048(1.0025–1.0070)*	1.0046(1.0032–1.0061)*	0.9960(0.9943–0.9977)*	1.0174(1.0104–1.0244)*	1.0145(1.0106–1.0185)*	2.3084(1.8483–2.8829)*
10	1.0028(1.0010–1.0047)*	1.0026(1.0004–1.0049)*	1.0031(1.0016–1.0045)*	0.9958(0.9941–0.9975)*	1.0058(0.9987–1.0129)	1.0106(1.0066–1.0145) *	1.7539(1.4017–2.1946)*

*p < 0.05.

**Table 8:** Relative risks (RRs) and 95% confidence intervals (CI) for association between air pollutant concentration and childhood asthma visits for over 11 years old children in different lag days in Nanjing from 2013 to 2021.

Lag days/d	AQI	PM_2.5_	PM_10_	O_3_	SO_2_	NO_2_	CO
0	0.9984(0.9935–1.0033)	1.0000(0.9942–1.0058)	0.9981(0.9942–1.0021)	0.9924(0.9870–0.9978)*	0.9881(0.9680–1.0087)	1.0065(0.9955–1.0176)	1.0166(0.5705–1.8117)
1	1.0057(1.0009–1.0105)*	1.0080(1.0023–1.0137)*	1.0042(1.0003–1.0080)*	0.9861(0.9812–0.9911)*	1.0145(0.9956–1.0338)	1.0211(1.0103–1.0320)*	2.2359(1.2633–3.9574)*
2	1.0001(0.9952–1.0049)	1.0007(0.9948–1.0065)	1.0006(0.9967–1.0045)	0.9858(0.9811–0.9905)*	0.9931(0.9745–1.0121)	1.0145(1.0039–1.0252)*	1.2276(0.6860–2.1967)
3	0.9960(0.9912–1.0009)	0.9959(0.9900–1.0018)	0.9972(0.9933–1.0011)	0.9850(0.9804–0.9896)*	0.9899(0.9713–1.0088)	1.0044(0.9939–1.0151)	0.5160(0.2848–0.9350)*
4	0.9930(0.9880–0.9979)*	0.9932(0.9873–0.9992)*	0.9936(0.9897–0.9976)*	0.9980(0.9834–0.9926)*	0.9853(0.9667–1.0042)	0.9959(0.9853–1.0065)	0.3493(0.1912–0.6382)*
5	0.9924(0.9874–0.9974)*	0.9917(0.9857–0.9977)*	0.9932(0.9893–0.9972)*	0.9864(0.9818–0.9910)*	0.9860(0.9673–1.0050)	0.9949(0.9844–1.0056)	0.4096(0.2237–0.7501)*
6	0.9945(0.9896–0.9995)*	0.9934(0.9874–0.9994)*	0.9951(0.9912–0.9991)*	0.9904(0.9858–0.9950)*	1.0093(0.9906–1.0284)	0.9965(0.9859–1.0072)	0.8532(0.4684–1.5541)
7	1.0008(0.9959–1.0057)	1.0017(0.9958–1.0077)	0.9976(0.9937–1.0015)	0.9948(0.9902–0.9995)*	0.9894(0.9707–1.0085)	0.9822(0.9717–0.9929)*	1.0985(0.6039–1.9983)
8	1.0067(1.0018–1.0116)*	1.0100(1.0041–1.0160)*	1.0032(0.9993–1.0071)	0.9925(0.9879–0.9971)*	1.0048(0.9861–1.0238)	0.9949(0.9844–1.0056)	2.4842(1.3756–4.4861)*
9	0.9998(0.9949–1.0048)	1.0011(0.9951–1.0071)	1.0001(0.9962–1.0041)	0.9959(0.9914–1.0005)*	1.0042(0.9855–1.0233)	0.9907(0.9801–1.0013)	1.6327(0.8993–2.9644)
10	1.0000(0.9951–1.0049)	1.0005(0.9946–1.0066)	0.9994(0.9955–1.0033)	0.9927(0.9881–0.9973)*	0.9811(0.9624–1.0002)	0.9838(0.9733–0.9945)*	0.7867(0.4297–1.4404)

*p < 0.05.

When stratified by age, the effects of air pollutants on daily childhood asthma visits showed some differences compared to the total population. However, the increase in CO concentration still had the most significant impact on the number of visits across all age groups. For children aged 0–5 years, the highest lag effect was observed for SO_2_ on lag day 2. The effects of AQI and other pollutants, including PM_2.5_, PM_10_, and NO_2_, in this age group followed a similar pattern to that of the total population, reaching their maximum impact on lag day 1, with a secondary peak on lag day 8, while the maximum impact of CO was observed on lag day 1, with a secondary peak on lag day 2. Meanwhile, O_3_ reached its maximum impact on lag day 10. In children aged 6–11 years, the largest lag effects of SO_2_ and NO_2_ followed a pattern similar to the total population, with maximum impact occurring on lag day 1. However, for AQI, PM_2.5_, PM_10_, O_3_ and CO, the most significant effects were observed on lag day 8. For children over 11 years, the largest lag effects for PM_10_ and NO_2_ mirrored those of the total population, peaking on lag day 1. However, the largest effects for AQI, PM_2.5_, and CO were similar to the pattern seen in children aged 6–11 years, with their maximum impact on lag day 8. O_3_ reached its maximum impact on lag day 9. Interestingly, the effect of SO_2_ on asthma visits in children over 11 years was weakly positive and not statistically significant.

When the concentrations of PM_2.5_, PM_10_, and NO_2_ increased by 10 ug/m^3^, the daily visits for children aged 0–5 years with asthma increased by 1.63% (95% CI: 1.0146–1.0181), 1.27% (95% CI: 1.0116–1.0139), and 5.12% (95% CI: 1.0479–1.0545) on lag day 1, respectively, all showing statistical significance. Additionally, a 10 ug/m^3^ increase in SO_2_ concentration was associated with a 6.84% increase in daily asthma visits (95% CI: 1.0629–1.0740) on lag day 2, with statistical significance.

For children aged 6–11 years, a 10 ug/m^3^ increase in PM_2.5_ and PM_10_ concentrations was linked to a 0.98% increase (95% CI: 1.0075–1.0120) and a 0.65% increase (95% CI: 1.0051–1.0080) in daily asthma visits on lag day 8, respectively, both statistically significant. Additionally, when SO_2_ and NO_2_ concentrations increased by 10 ug/m^3^, daily asthma visits in this age group increased by 3.84% (95% CI: 1.0314–1.0455) and 3.42% (95% CI: 1.0303–1.0382) on lag day 1, respectively, showing statistical significance.

For children aged over 11 years, a 10 ug/m^3^ increase in PM_2.5_ concentration resulted in a 1.00% increase in daily asthma visits (95% CI: 1.0041–1.0160) on lag day 8, which was statistically significant. Additionally, a 10 ug/m^3^ increase in PM_10_ and NO_2_ concentrations was associated with a 0.42% (95% CI: 1.0003–1.0080) and 2.11% increase (95% CI: 1.0103–1.0320) in daily asthma visits on lag day 1, respectively, with statistical significance.

#### 3.3.4 Lag effect by season

Tables 9–12 present the association between AQI, air pollutant concentrations, and the number of childhood asthma visits stratified by season in Nanjing from 2013 to 2021. The analysis reveals distinct seasonal lag patterns in the impact of air pollutants on daily childhood asthma visits. In spring, autumn, and winter, an increase in CO concentration had the most significant impact on the number of visits. However, in summer, an increase in CO concentration did not lead to a rise in asthma visits.

**Table 9:** Relative risks (RRs) and 95% confidence intervals (CI) for association between air pollutant concentration and childhood asthma visits in spring in different lag days in Nanjing from 2013 to 2021.

Lag days/d	AQI	PM_2.5_	PM_10_	O_3_	SO_2_	NO_2_	CO
0	0.9988(0.9956–1.0020)	1.0031(0.9990–1.0072)	0.9977(0.9955–0.9999)*	0.9973(0.9941–1.0005)	1.0625(1.0497–1.0755)*	1.0023(0.9962–1.0085)	1.5080(1.0386–2.1897)*
1	0.9998(0.9967–1.0028)	1.0010(0.9969–1.0051)	0.9989(0.9969–1.0010)	0.9966(0.9940–0.9993)*	1.0332(1.0217–1.0448)*	1.0066(1.0006–1.0126)*	1.2729(0.8798–1.8418)
2	0.9994(0.9965–1.0024)	0.9989(0.9949–1.0030)	0.9999(0.9980–1.0019)	0.9938(0.9914–0.9962)*	1.0359(1.0249–1.0471)*	1.0097(1.0038–1.0156)*	1.9547(1.3606–2.8083)*
3	0.9999(0.9970–1.0029)	0.9977(0.9937–1.0017)	1.0001(0.9981–1.0020)	0.9922(0.9899–0.9946)*	1.0286(1.0177–1.0396)*	0.9989(0.9932–1.0046)	1.7450(1.2204–2.4951)*
4	0.9992(0.9962–1.0023)	0.9948(0.9907–0.9989)*	0.9996(0.9975–1.0016)	0.9975(0.9953–0.9998)*	1.0237(1.0127–1.0349)*	0.9920(0.9863–0.9977)*	0.7530(0.5238–1.0824)
5	1.0018(0.9988–1.0049)	1.0003(0.9962–1.0045)	0.9983(0.9963–1.0003)	0.9986(0.9964–1.0008)	1.0130(1.0020–1.0241)*	0.9951(0.9893–1.0008)	0.9435(0.6564–1.3563)
6	1.0099(1.0058–1.0140)*	1.0099(1.0058–1.0140)*	1.0028(1.0009–1.0048)*	0.9968(0.9946–0.9991)*	1.0278(1.0166–1.0391)*	1.0049(0.9991–1.0107)	3.0943(2.1569–4.4393)*
7	1.0057(1.0016–1.0099)	1.0057(1.0016–1.0099)*	1.0017(0.9997–1.0037)	0.9997(0.9975–1.0019)	1.0442(1.0327–1.0559)*	1.0072(1.0013–1.0131)*	2.5263(1.7448–3.6579)*
8	1.0140(1.0110–1.0170)*	1.0180(1.0138–1.0222)*	1.0088(1.0068–1.0108)*	1.0048(1.0026–1.0070)*	1.0700(1.0583–1.0817)*	1.0143(1.0084–1.0203)*	2.4065(1.6633–3.4819)*
9	1.0068(1.0039–1.0098)*	1.0091(1.0050–1.0133)*	1.0064(1.0044–1.0083)*	1.0035(1.0013–1.0057)*	1.0619(1.0506–1.0734)*	1.0204(1.0145–1.0264)*	2.4103(1.6763–3.4657)*
10	1.0005(0.9975–1.0034)	1.0011(0.9970–1.0052)	1.0012(0.9993–1.0032)	1.0015(0.9993–1.0037)	1.0332(1.0221–1.0444)*	1.0062(1.0005–1.0120)*	2.9437(2.0622–4.2018)*

*p < 0.05.

**Table 10:** Relative risks (RRs) and 95% confidence intervals (CI) for association between air pollutant concentration and childhood asthma visits in summer in different lag days in Nanjing from 2013 to 2021.

Lag days/d	AQI	PM_2.5_	PM_10_	O_3_	SO_2_	NO_2_	CO
0	0.9999(0.9959–1.0039)	0.9994(0.9946–1.0044)	0.9972(0.9937–1.0008)	1.0054(1.0029–1.0079)*	0.9231(0.9050–0.9415)*	0.9942(0.9844–1.0042)	0.5166(0.3274–0.8152)*
1	1.0018(0.9982–1.0055)	1.0000(0.9953–1.0047)	0.9997(0.9965–1.0030)	1.0030(1.0010–1.0050)*	0.9736(0.9562–0.9913)*	1.0017(0.9920–1.0115)	0.5023(0.3204–0.7873)*
2	1.0016(0.9980–1.0052)	0.9994(0.9947–1.0041)	0.9983(0.9950–1.0015)	1.0018(0.9999–1.0037)	0.9472(0.9310–0.9637)*	1.0002(0.9907–1.0099)	0.3839(0.2459–0.5995)*
3	0.9949(0.9914–0.9985)*	0.9928(0.9882–0.9975)*	0.9958(0.9926–0.9990)*	1.0018(1.0000–1.0036)	0.9514(0.9353–0.9677)*	1.0091(0.9997–1.0186)	0.2963(0.1903–0.4615)*
4	0.9973(0.9938–1.0008)	0.9972(0.9926–1.0018)	0.9949(0.9918–0.9981)*	1.0019(1.0001–1.0036)*	0.9463(0.9309–0.9619)*	1.0067(0.9973–1.0161)	0.5558(0.3571–0.8652)*
5	1.0030(0.9995–1.0066)	1.0046(0.9999–1.0092)	1.0008(0.9976–1.0040)	1.0029(1.0011–1.0046)*	0.9365(0.9216–0.9516)*	1.0115(1.0019–1.0211)*	1.1683(0.7485–1.8235)
6	1.0037(1.0000–1.0073)	1.0030(0.9982–1.0078)	1.0003(0.9970–1.0036)	1.0053(1.0035–1.0071)*	0.9582(0.9429–0.9737)*	1.0124(1.0026–1.0222)*	0.5776(0.3691–0.9038)*
7	1.0075(1.0038–1.0112)*	1.0036(0.9988–1.0084)	1.0001(1.0067–1.0034)	1.0061(1.0042–1.0079)*	0.9378(0.9226–0.9534)*	0.9900(0.9804–0.9997)*	0.4550(0.2916–0.7100)*
8	1.0051(1.0014–1.0087)*	1.0048(1.0001–1.0096)*	1.0019(0.9986–1.0051)	1.0064(1.0046–1.0082)*	0.9563(0.9409–0.9720)*	1.0100(1.0004–1.0198)*	0.6555(0.4203–1.0223)
9	1.0070(1.0035–1.0106)*	1.0093(1.0047–1.0140)*	1.0052(1.0020–1.0084)*	1.0043(1.0025–1.0061)*	0.9643(0.9489–0.9799)*	1.0322(1.0225–1.0419)*	1.3773(0.8892–2.1331)
10	1.0005(0.9970–1.0040)	0.9982(0.9937–1.0027)	0.9989(0.9957–1.0020)	1.0031(1.0013–1.0049)*	0.9633(0.9481–0.9788)*	1.0190(1.0095–1.0285)*	0.4856(0.3156–0.7472)*

*p < 0.05.

**Table 11:** Relative risks (RRs) and 95% confidence intervals (CI) for association between air pollutant concentration and childhood asthma visits in autumn in different lag days in Nanjing from 2013 to 2021.

Lag days/d	AQI	PM_2.5_	PM_10_	O_3_	SO_2_	NO_2_	CO
0	1.0093(1.0068–1.0118)*	1.0084(1.0054–1.0115)*	1.0080(1.0062–1.0098)*	1.0021(0.9990–1.0052)	1.0142(1.0020–1.0265)*	1.0014(0.9965–1.0063)	1.8410(1.4867–2.2797)*
1	1.0136(1.0111–1.0160)*	1.0165(1.0134–1.0195)*	1.0092(1.0074–1.0110)*	0.9979(0.9853–1.0005)	1.0392(1.0283–1.0503)*	1.0152(1.0106–1.0198)*	2.8768(2.3276–3.5555)*
2	1.0070(1.0046–1.0095)*	1.0083(1.0053–1.0113)*	1.0056(1.0038–1.0074)*	1.0034(1.0011–1.0058)*	1.0346(1.0238–1.0456)*	1.0155(1.0110–1.0200)*	1.8303(1.4767–2.2687)*
3	1.0071(1.0047–1.0095)*	1.0102(1.0072–1.0132)*	1.0060(1.0042–1.0077)*	0.9985(0.9963–1.0008)	1.0318(1.0209–1.0428)*	1.0240(1.0195–1.0285)*	1.3032(1.0485–1.6197)*
4	1.0038(1.0013–1.0062)*	1.0065(1.0035–1.0095)*	1.0026(1.0008–1.0043)*	1.0036(1.0015–1.0058)*	1.0372(1.0265–1.0480)*	1.0167(1.0123–1.0212)*	1.1303(0.9072–1.4081)*
5	1.0017(0.9993–1.0042)	1.0007(0.9977–1.0037)	1.0028(1.0011–1.0046)	1.0024(1.0003–1.0045)*	1.0443(1.0338–1.0550)*	1.0101(1.0057–1.0146)*	0.9634(0.7716–1.2029)
6	0.9983(0.9959–1.0007)	0.9936(0.9905–0.9966)*	1.0002(0.9985–1.0019)	0.9990(0.9969–1.0012)	1.0243(1.0137–1.0350)*	0.9974(0.9930–1.0018)	1.3396(1.0723–1.6736)*
7	0.9964(0.9940–0.9989)*	0.9926(0.9895–0.9957)*	0.9983(0.9966–1.0001)	1.0047(1.0026–1.0067)*	0.9937(0.9830–1.0044)	0.9832(0.9789–0.9876)*	1.9804(1.5850–2.4744)*
8	1.0056(1.0032–1.0081)*	1.0048(1.0017–1.0079)*	1.0036(1.0019–1.0054)*	1.0024(1.0004–1.0044)*	1.0098(0.9991–1.0207)	0.9958(0.9915–1.0002)	3.2184(2.5811–4.0129)
9	0.9990(0.9965–1.0015)	0.9967(0.9936–0.9999)*	1.0015(0.9997–1.0033)	1.0015(0.9995–1.0035)	1.0320(1.0212–1.0428)*	0.9992(0.9948–1.0036)	2.3084(1.8483–2.8829)
10	1.0012(0.9988–1.0037)	1.0016(0.9985–1.0047)	1.0014(0.9997–1.0032)	1.0014(0.9994–1.0034)	1.0033(0.9928–1.0140)	1.0071(1.0027–1.0115)*	1.7539(1.4017–2.1946)*

*p < 0.05.

**Table 12:** Relative risks (RRs) and 95% confidence intervals (CI) for association between air pollutant concentration and childhood asthma visits in winter in different lag days in Nanjing from 2013 to 2021.

Lag days/d	AQI	PM_2.5_	PM_10_	O_3_	SO_2_	NO_2_	CO
0	1.0074(1.0057–1.0091)*	1.0082(1.0062–1.0102)*	1.0087(1.0073–1.0102)*	0.9496(0.9460–0.9532)*	1.0443(1.0377–1.0509)*	1.0558(1.0517–1.0599)*	2.9343(2.3670–3.6375)*
1	1.0154(1.0138–1.0169)*	1.0172(1.0154–1.0191)*	1.0157(1.0143–1.0171)*	0.9489(0.9454–0.9524)*	1.0672(1.0610–1.0735)*	1.0729(1.0688–1.0770)*	10.8406(8.8163–13.3295)*
2	1.0120(1.0105–1.0136)*	1.0127(1.0109–1.0145)*	1.0121(1.0108–1.0135)*	0.9441(0.9406–0.9476)*	1.0589(1.0527–1.0652)*	1.0515(1.0476–1.0553)*	4.3323(3.5454–5.2939)*
3	1.0111(1.0096–1.0127)*	1.0122(1.0104–1.0140)*	1.0111(1.0098–1.0125)*	0.9509(0.9474–0.9544)*	1.0492(1.0429–1.0554)*	1.0436(1.0398–1.0474)*	2.8844(2.3614–3.5233)*
4	1.0058(1.0042–1.0074)*	1.0067(1.0048–1.0086)*	1.0066(1.0053–1.0080)*	0.9516(0.9481–0.9552)*	1.0387(1.0324–1.0451)*	1.0421(1.0383–1.0458)*	2.6750(2.1833–3.2775)*
5	1.0029(1.0013–1.0046)*	1.0027(1.0009–1.0046)*	1.0036(1.0022–1.0050)*	0.9625(0.9589–0.9660)*	1.0174(1.0108–1.0240)*	1.0360(1.0323–1.0398)*	1.7121(1.3926–2.1048)*
6	1.0020(1.0004–1.0037)*	1.0014(0.9995–1.0033)	1.0031(1.0017–1.0045)*	0.9644(0.9609–0.9679)*	1.0515(1.0447–1.0584)*	1.0424(1.0387–1.0462)*	2.0905(1.6996–2.5713)*
7	1.0087(1.0071–1.0103)*	1.0094(1.0075–1.0112)*	1.0093(1.0079–1.0106)*	0.9782(0.9747–0.9817)*	1.0614(1.0549–1.0681)*	1.0396(1.0359–1.0433)*	4.6013(3.7495–5.6466)*
8	1.0118(1.0102–1.0134)*	1.0136(1.0118–1.0155)*	1.0126(1.0112–1.0139)*	0.9680(0.9646–0.9715)*	1.0491(1.0429–1.0554)*	1.0424(1.0386–1.0461)*	7.7813(6.3734–9.5003)*
9	1.0069(1.0053–1.0085)*	1.0077(1.0059–1.0096)*	1.0070(1.0056–1.0084)*	0.9767(0.9733–0.9801)*	1.0151(1.0088–1.0214)*	1.0169(1.0132–1.0205)*	2.1653(1.7667–2.6538)*
10	1.0079(1.0063–1.0096)*	1.0088(1.0069–1.0107)*	1.0083(1.0069–1.0097)*	0.9818(0.9783–0.9853)*	1.0187(1.0123–1.0252)*	1.0078(1.0041–1.0115)*	2.0931(1.7025–2.5734)*

*p < 0.05.

In spring, the maximum impact on childhood asthma visits occurred on lag day 8 for AQI, PM_2.5_, PM_10_, O_3_ and SO_2_. The largest effects of NO_2_ and CO were observed on lag day 9 and lag day 6, respectively. When the concentrations of PM_2.5_, PM_10,_ and SO_2_ increased by 10 ug/m^3^, the number of daily visits for children with asthma increased by 1.80% (95% CI: 1.0138–1.0222), 0.88% (95% CI: 1.0068–1.0108), and 7.00% (95% CI: 1.0583–1.0817), respectively, on lag day 8, with statistical significance. Additionally, a 10 ug/m^3^ increase in NO_2_ concentration was associated with a 2.04% (95% CI: 1.0145–1.0264) increase in daily asthma visits on lag day 9.

During the summer, the maximum impact on childhood asthma visits was observed on lag day 9 for PM_2.5_, PM_10_ and NO_2_, on lag day 6 for AQI, and on lag day 8 for O_3_. However, SO_2_ continued to exhibit its maximum impact on lag day 1. A 10 ug/m^3^ increase in PM_2.5_, PM_10_, and NO_2_ concentrations resulted in significant increases in daily childhood asthma visits by 0.93% (95% CI: 1.0047–1.0140), 0.52% (95% CI: 1.0020–1.0084), and 3.22% (95% CI: 1.0225–1.0419), respectively, on lag day 9. No statistically significant increase in asthma visits was observed with a 10 ug/m^3^ increase in SO_2_ concentration during the summer season.

In autumn, the maximum impact on childhood asthma visits occurred on lag day 1 for AQI, PM_2.5_, and PM_10_, which mirrors the pattern observed in the total population. However, the maximum impact of O_3_ was observed on lag day 7, SO_2_ on lag day 5, NO_2_ on lag day 3, and CO on lag day 8. When the concentrations of PM_2.5_ and PM_10_ increased by 10 ug/m^3^, the number of daily asthma visits increased by 1.65% (95% CI: 1.0134–1.0195) and 0.92% (95% CI: 1.0074–1.0110), respectively, on lag day 1, with statistical significance. An increase of 10 ug/m^3^ in SO_2_ concentration was associated with a 4.43% (95% CI: 1.0338–1.0550) increase in asthma visits on lag day 5, and a similar increase in NO_2_ concentration led to a 2.40% (95% CI: 1.0195–1.0285) increase on lag day 3.

In winter, the pattern of air pollutant effects on childhood asthma visits resembled that observed in the total population. The maximum impact was observed on lag day 1 for AQI, PM_2.5_, PM_10_, SO_2_, NO_2_, and CO. A 10 ug/m^3^ increase in PM_2.5_, PM_10_, SO_2_, and NO_2_ concentrations was associated with statistically significant increases in daily asthma visits by1.72% (95% CI: 1.0154–1.0191), 1.57% (95% CI: 1.0143–1.0171), 6.72% (95% CI: 1.0610–1.0735), and 7.29% (95% CI: 1.0688–1.0770), respectively, on lag day 1.

## 4 Discussion

The present study assessed the effects of air pollutants on childhood asthma visits in Nanjing from 2013 to 2021, revealing a positive correlation between childhood asthma visits and the concentrations of AQI, PM_2.5_, PM_10_, SO_2_, NO_2_, and CO. The findings suggest that increases in these pollutants are associated with an elevated number of childhood hospital visits due to asthma exacerbations. However, no significant association was found between childhood asthma visits and O_3_ concentrations, a result consistent with some studies both domestically and internationally (15, 24, 25). The maximum effects of AQI and other pollutants, such as PM_2.5_, PM_10_, SO_2_, NO_2_, and CO, on daily childhood asthma visits in the total population were observed on lag day 1, which differs from reports in other cities (15, 26, 27). This discrepancy might be attributed to differences in the composition of air pollutants across cities and the influence of climatic factors, resulting in varying impacts on childhood asthma visits.

### 4.1 Gender differences

When stratifying the analysis by gender, it was observed that PM_2.5_ and PM_10_ had a significantly greater impact on females than males, in terms of both lagged effects and the increased number of daily childhood asthma visits when concentrations rose by 10 ug/m^3^. This finding is similar to results from studies conducted in Shenyang (13), although some other studies have found gender to be less significant (28, 29). Similar, CO had the greatest impact on female children aged 0–5 years (29), particularly in winter when CO was found to be a primary pollutant contributing to the observed increase in asthma visits.

### 4.2 Age-related effects

Age stratification revealed that the impact of air pollutants, including PM_2.5_, PM_10_, SO_2_, and NO_2_, was strongest among children aged 0–5 years, which aligns with the majority of studies in the field (30). However, a study conducted in Shanghai, geographically close to Nanjing, found that PM_2.5_ had a stronger impact on children aged 5–14 years compared to those aged 0–4 years. Additionally, with increasing age, both the duration of the lagged effect and the magnitude of the increase in daily asthma visits diminish. Notably, SO_2_ showed no significant impact on children over 11 years old. One possible explanation is that older children have relatively mature respiratory and immune systems, resulting in a milder response to air pollutants. Additionally, older children tend to engage in more indoor activities, reducing their exposure to outdoor air pollutants.

### 4.3 Seasonal variations and underlying factors

Seasonal stratification demonstrated that the effect of air pollutants on childhood asthma varies considerably. A 10 ug/m^3^ increase in PM_2.5_ and SO_2_ concentrations had the greatest impact on childhood asthma visits in spring, whereas PM_10_ and NO_2_ showed the most significant effects during winter. The most prolonged lagged effects were observed in winter, followed by spring, autumn, and summer. Notably, SO_2_ had no significant impact on childhood asthma visits during the summer.

The underlying reasons for these seasonal differences may be multifactorial. First, the composition of air pollutants likely varies by season. For example, in winter, the increased use of coal and biomass for heating results in higher concentrations of primary pollutants such as PM_2.5_, SO_2_, and CO (31). Conversely, during summer, increased solar radiation promotes photochemical reactions, leading to elevated O_3_ levels while reducing primary pollutants. Additionally, meteorological factors such as wind patterns, atmospheric inversions, and air pressure may influence pollutant dispersion and accumulation. During winter, stable atmospheric conditions with frequent temperature inversions can trap pollutants near ground level, prolonging exposure time and exacerbating asthma symptoms (32).

Moreover, children’s behavior and activity patterns also vary seasonally, influencing their exposure and susceptibility to air pollution. In winter, children may spend more time indoors, reducing overall exposure to outdoor air pollutants. However, indoor pollutants such as CO from heating systems may increase. In summer, children generally spend more time outdoors, potentially increasing their exposure to ambient air pollutants but also benefiting from improved air dispersion conditions. This shift in exposure may partly explain the seasonal differences observed in asthma exacerbations (33).

Interestingly, the study found a negative correlation between childhood asthma visits and CO concentrations in summer, but a positive correlation in other seasons. This pattern is consistent with other studies (34, 35). It is possible that in summer, higher atmospheric convection and air mixing lead to the rapid dispersion of CO, diminishing its impact to asthma. Conversely, winter conditions facilitate pollutant accumulation, amplifying CO’s harmful effects on respiratory health (36). This seasonal variation suggests a need to consider specific mitigation strategies tailored to each season to reduce the impact of air pollution on childhood asthma.

### 4.4 Mechanistic considerations, synergistic effects, and policy implications

Compared to inhalable particulate matter (PM_10_), PM_2.5_ has a smaller particle size, a larger surface area, and a greater capacity for allergen adsorption, along with a longer atmospheric lifespan. As a result, PM_2.5_ consistently exerts a greater impact on childhood asthma visits than PM_10_, regardless of gender, age, or season, a finding that aligns with previous studies (37–40).

Due to its smaller particle size distribution, PM_2.5_ can bypass the upper respiratory tract’s ciliary clearance mechanism, penetrate deep into the bronchial tree, and deposit in the alveolar region. It can further cross the air-blood barrier, leading to irreversible bioaccumulation and triggering oxidative stress and inflammatory responses in the airway epithelium. This process induces the release of pro-inflammatory cytokines and chemokines (41, 42), which in turn recruit immune cells such as neutrophils and eosinophils to the lungs, amplifying the inflammatory cascade (43, 44). Inhalation of PM_2.5_ can also alter immune responses by activating toll-like receptors (TLRs), inducing airway hyperresponsiveness, and impairing the airway’s ability to clear pathogens, thereby increasing the risk of respiratory infections and asthma exacerbations (45, 46). These effects are particularly pronounced in vulnerable populations, such as children and individuals with pre-existing respiratory diseases. Furthermore, the high content of heavy metals and polycyclic aromatic hydrocarbons (PAHs) in PM_2.5_ is more likely to induce airway inflammation (47).

The observed significant rise in asthma incidence with increased NO_2_ concentration might be explained by children’s vulnerability due to their developing respiratory systems and higher physical activity levels, leading to increased air intake. NO_2_ primarily originates from vehicle emissions, industrial discharges, and fossil fuel combustion. Research has indicated that children carrying specific glutathione S-transferase pi gene alleles (rs113872 or rs1695) may be at higher risk of asthma attacks when exposed to NO_2_ (48). It can interact with other pollutants, particularly PM_2.5_, to form secondary fine particulate matter, further worsening respiratory symptoms and inflammation. Additionally, NO_2_ may directly damage respiratory epithelial cells, disrupt lung function, and increase sensitization to allergens. To reduce childhood asthma incidence and mitigate the adverse effects of NO_2_, effective measures, such as promoting clean energy, implementing vehicle emission restrictions, and enhancing industrial pollution control, are needed, particular in densely populated urban and industrial areas.

Among the four air pollutants, SO_2_ has the most significant effect on childhood asthma across various demographic groups and seasons. However, unlike other pollutants, the impact of SO_2_ is not significant in children over 11 years of age or during the summer. Similarly, SO_2_, primarily from industrial emissions and vehicle exhaust, can exacerbate asthma symptoms in children, including shortness of breath, coughing, and chest tightness. Although the mechanism is not fully understood, SO_2_ may induce inflammatory responses or cause bronchospasm in the respiratory tract. It can also cause oxidative stress and potentially impact asthma by influencing the autonomic nervous system. However, emerging evidence suggests that SO_2_ may act synergistically with PM_2.5_ to increase airway oxidative stress and disrupt the epithelial barrier, potentially amplifying asthma risk (49). Additionally, SO_2_ can also react with PM_2.5_ or NO_2_ to form secondary sulfate aerosols, which have a more potent impact on lung function decline and asthma exacerbations (50).

The impact of O_3_ concentration on childhood asthma differs from other pollutants. After stratification by gender and age, O_3_ showed a negative correlation with childhood asthma visits. When considering seasonal variations, a weak positive correlation was noted in spring, summer, and autumn, while a negative correlation persisted in winter. This suggests that the relationship between O_3_ and childhood asthma is complex and may be influenced by various factors such as other environmental conditions and individual susceptibility. Research suggests that O_3_ exposure may enhance the sensitivity of airways to PM2.5, resulting in a more pronounced inflammatory response.

Our results align with previous research demonstrating a strong association between air pollution and childhood asthma exacerbations. Studies conducted in cities such as Hangzhou (51), Shanghai (21), and Chongqing (15) have reported a significant association between PM_2.5_ and asthma-related hospital visits, which is consistent with our findings. However, in Shenyang, the main pollutants identified were PM_10_, CO, and O_3_. Additionally, a study in Hangzhou found a significant correlation between NO_2_, SO_2_, and childhood asthma hospitalization rates. A notable difference in our study is the negative correlation between CO concentrations and childhood asthma visits in the summer, while in other seasons, the correlation was positive. This seasonal variation has not been reported in studies from other regions of China. This difference may be attributed to regional variations in emission sources, meteorological conditions, and pollutant composition. In southern cities like Nanjing, higher humidity and temperatures may promote the formation of secondary air pollutants, thereby amplifying the health effects of CO exposure. The threshold levels at which air pollutants trigger adverse health effects can vary by region, likely due to differences in population susceptibility, healthcare accessibility, and air quality standards. Future research should further investigate these regional disparities, incorporating multi-city and international comparative analyses to refine exposure-risk assessments and inform targeted intervention strategies.

While air pollution is a recognized trigger and exacerbating factor for childhood asthma, it may not be the sole driver of the rising asthma prevalence, especially considering that air quality has improved in many regions including China. Emerging evidence suggests that other environmental factors, such as indoor and environmental microbiome changes, gut microbiota dysbiosis, and persistent organic pollutants (POPs) exposure, may also contribute to the increased asthma burden (29). Recent studies also highlight that air pollution can alter the indoor microbiome, further affecting asthma outcomes (52).

Our findings underscore the complex interplay between air pollutants, demographic factors, and seasonal variations in affecting childhood asthma, highlighting the urgent need for targeted interventions to mitigate its impact. Given the heightened pollution levels during certain seasons, particularly winter, policymakers should strengthen air quality regulations by enforcing stricter limits on PM_2.5_, NO_2_, and CO emissions, especially in industrial and high-traffic urban areas. Effective pollution control measures, such as promoting cleaner energy sources, restricting vehicle emissions during peak pollution periods, and expanding green spaces, are essential to improving air quality. Additionally, enhancing public awareness through real-time air quality alerts and providing guidance for vulnerable populations, such as young children and those in heavily polluted areas, can help minimize exposure risks. Improving indoor air quality through air purifiers and ventilation systems in schools and households can further protect children from airborne pollutants. Moreover, developing early warning systems that utilize real-time pollution data can help predict asthma exacerbation risks and enable proactive healthcare interventions. While these measures are crucial for reducing asthma-related hospital visits and improving respiratory health in children, future research should explore the role of pollutant interactions, environmental microbiome changes, and persistent organic pollutants (POPs) exposure in driving the continued rise of childhood asthma prevalence despite improvements in air quality.

## 5 Limitations

This study has several limitations. First, the data on air pollution and childhood asthma visits were collected in Nanjing, which may limit the generalizability of the findings to other regions with different climatic conditions, pollution sources, and healthcare access. Second, the study only used air quality data from fixed monitoring stations, which may not accurately represent individual exposure levels, especially since children may have different activity patterns, including indoor versus outdoor time. Third, the study relied on hospital visit records to identify asthma cases, potentially overlooking mild cases managed at home or in outpatient clinics. Additionally, we did not account for other potential confounding factors, such as viral infections, indoor air pollutants, or socioeconomic status, which could also influence asthma exacerbations. Lastly, the study primarily focused on the short-term lag effects of air pollution on acute asthma exacerbations in children, however, these acute exacerbations may contribute to the long-term progression of asthma (53–55). Therefore, the short-term lag effects observed in our study may reflect a broader cumulative impact of long-term exposure, which warrants further longitudinal investigations. Future research should consider these aspects to provide a more comprehensive understanding of the relationship between air pollution and childhood asthma.

## 6 Conclusion

This study demonstrates a significant positive correlation between air pollution and the number of childhood asthma visits in Nanjing from 2013 to 2021. Increases in concentrations of pollutants, such as PM_2.5_, PM_10_, SO_2_, NO_2_, and CO, were associated with an elevated risk of asthma exacerbations, particularly among young children aged 0–5 years and during specific seasons like spring and winter. Notably, SO₂ and PM_2.5_ had the most substantial impacts on childhood asthma, with effects varying based on age, gender, and season. Conversely, O₃ concentration showed a negative or weak positive correlation with childhood asthma visits, differing from other pollutants.

The study also reveals that younger children, especially females, are more vulnerable to the adverse effects of air pollution. Additionally, seasonal variations highlight that certain pollutants, such as SO₂, have minimal impact in the summer, possibly due to atmospheric dispersion and reduced outdoor activities. These findings underscore the importance of targeted interventions to reduce air pollution exposure, particularly for susceptible groups, and the need for stricter air quality control measures during seasons with higher pollution impacts.

This study contributes to a growing body of evidence linking air pollution to childhood asthma exacerbations. Future studies should focus on individual exposure assessments, long-term effects, and additional confounding factors to develop comprehensive strategies for mitigating the health impacts of air pollution on children.

## Data Availability

The original contributions presented in the study are included in the article/supplementary material, further inquiries can be directed to the corresponding authors.
